# Molecular mechanisms underlying the lifespan and healthspan benefits of dietary restriction across species

**DOI:** 10.3389/fgene.2026.1771707

**Published:** 2026-01-30

**Authors:** Jialin Fan, Yunpeng Xu

**Affiliations:** 1 Rutgers Cancer Institute, Rutgers, The State University of New Jersey, New Brunswick, NJ, United States; 2 Department of Molecular Biology and Biochemistry, Rutgers University, New Brunswick, NJ, United States

**Keywords:** aging, diet restriction, healthspan, lifespan, longevity, mTOR

## Abstract

Dietary restriction (DR), defined as reduced caloric intake or selective limitation of specific nutrients without malnutrition, is one of the most robust interventions known to extend lifespan and healthspan across species. Studies from yeast to mammals demonstrate that DR elicits conserved genetic, transcriptional, and epigenetic programs that promote cellular maintenance and stress resistance. At the molecular level, DR engages evolutionarily conserved nutrient-sensing pathways, including insulin/IGF-1 signaling (IIS), the mechanistic target of rapamycin (mTOR), AMP-activated protein kinase (AMPK), and NAD^+^-dependent sirtuins, which converge on key transcription factors (TFs) and transcriptional coactivators (TCs) to coordinate metabolic and longevity-associated gene expression. Downstream, these pathways enhance autophagy and proteostasis, remodel mitochondrial function and redox balance, reshape immune and inflammatory networks, and induce epigenetic and transcriptional reprogramming. Recent work further highlights amino acid–specific sensing mechanisms, endocrine mediators such as fibroblast growth factor 21 (FGF21), the gut microbiome, circadian regulators, and nuclear pore–associated transcriptional plasticity as integral components of DR responses. Importantly, the physiological outcomes of DR are context dependent and influenced by genetic background, sex, age at intervention, and the type and duration of restriction. In this review, we summarize current knowledge on the genetic and molecular architecture underlying DR-induced longevity and health benefits across species, discuss implications for aging-related diseases, and outline future directions toward precision nutrition and safe translational strategies.

## 1 Introduction

Aging is characterized by a progressive decline in physiological integrity, reduced stress resilience, and increased susceptibility to chronic diseases (Lopez-Otin et al., 2023). Among numerous genetic, pharmacological, and lifestyle interventions examined over the past decades, dietary restriction (DR) remains the most robust and evolutionarily conserved strategy for extending lifespan and improving healthspan. Originally described in rodents nearly a century ago, the beneficial effects of reduced nutrient intake have since been validated in a wide range of organisms, including yeast, nematodes, flies, and mammals (Wu et al., 2022). While often used interchangeably, it is critical to distinguish between different nutritional interventions to avoid conceptual overlap. Caloric restriction (CR) typically refers to a chronic reduction in total calorie intake (usually 20%–40%) without malnutrition. In contrast, Chronic Dietary Restriction (DR) is a broader term encompassing the restriction of specific macronutrients (amino acid restriction, protein restriction) regardless of total calorie count. Furthermore, long-term Fasting involves extended periods without food intake, triggering distinct periodic metabolic switches that differ from the continuous physiological adaptations induced by chronic CR or DR.

Genetic and transcriptomic studies have revealed that DR induces coordinated changes in gene expression, chromatin state, and metabolic wiring, leading to a systemic shift from anabolic growth toward cellular maintenance and stress resistance (Longo and Anderson, 2022; Wilson et al., 2021). Central to these are conserved nutrient-sensing pathways—such as insulin/IGF-1 signaling, the target of rapamycin (mTOR), AMP-activated protein kinase (AMPK), and NAD^+^-dependent sirtuins—that function as molecular hubs linking environmental cues to transcriptional and epigenetic regulation. These pathways regulate the activity of key transcription factors and transcriptional coactivators, thereby shaping long-term gene expression programs associated with longevity.

Growing evidence indicates that the qualitative aspects of diet, including macronutrient composition and individual amino acid availability, play a critical role in determining DR outcomes. Protein restriction and amino acid–specific interventions can reproduce many benefits of caloric restriction, highlighting the importance of nutrient-specific genetic sensing mechanisms. Moreover, endocrine signals, inter-organ communication, the gut microbiome, and emerging regulators such as microRNA (*mir-235*/miR-92, *mir-228*/miR-96, *mir-71*, *mir-80*) (Vora et al., 2013; Smith-Vikos et al., 2014; Xu et al., 2019), the nuclear pore complex further integrates DR signals at the organismal level (Zhou et al., 2025). While many DR-regulated microRNA mechanisms were first identified in *C. elegans*, emerging evidence suggests these pathways are partially conserved in mammals. However, given the increased tissue complexity and regulatory redundancy in higher organisms, direct translation remains challenging, underscoring the need for further mammalian validation.

Notably, many of the genetic pathways engaged by DR overlap with those implicated in aging-related diseases, including metabolic disorders, neurodegeneration, cardiovascular disease, and cancer. This overlap underscores the translational relevance of dissecting DR-induced genetic programs, while emphasizing the need to understand context-dependent effects influenced by genetic background, sex, age, and intervention timing (Wu et al., 2022; Madeo et al., 2019; Taylor et al., 2022; Tajan and Vousden, 2020; Kanarek et al., 2020; Brandhorst and Longo, 2019).

Importantly, the effects of dietary restriction (DR) on lifespan and healthspan are not uniform across studies or species. Accumulating evidence indicates that DR responses are highly context dependent, influenced by genetic background, baseline diet composition, age at intervention, sex, duration and severity of restriction, and environmental conditions (Forney et al., 2020; Liao et al., 2013; Regan et al., 2016; Vega et al., 2004). Moreover, DR does not operate as a linear intervention: while moderate restriction often elicits adaptive stress responses and longevity benefits, excessive or prolonged restriction can impair immune function, tissue repair, reproductive capacity, and skeletal integrity. These non-linear and species-specific effects underscore the need to move beyond descriptive pathway cataloging toward integrative frameworks that explain why DR is beneficial in some contexts but detrimental in others. Addressing this variability is essential for understanding the mechanisms of DR and for translating its benefits into safe and effective interventions in humans.

While macronutrient composition is the primary driver of dietary restriction (DR) benefits, the role of specific micronutrients remains an emerging field. Although current evidence for lifespan extension via broad micronutrient restriction is limited compared to caloric restriction (CR), ongoing research suggests that the targeted modulation of specific vitamins or minerals may yield significant breakthroughs in treating age-related pathologies.

Beyond summarizing individual nutrient-sensing pathways, this review aims to provide an integrative conceptual framework for understanding how dietary restriction (DR) exerts durable effects on lifespan and healthspan across species. It is well-recognized that DR activates a hierarchically organized transcriptional and epigenetic program downstream of conserved nutrient-sensing pathways, including insulin/IGF-1, mTOR, and AMPK–sirtuin signaling. In this framework, metabolic signals are translated into coordinated transcriptional outputs that are stabilized through chromatin remodeling and epigenetic mechanisms, thereby generating long-lasting physiological adaptations rather than transient metabolic responses. By emphasizing transcriptional integration, cross-species conservation, and potential forms of aging “memory”, this review seeks to bridge nutrient sensing, gene regulation, and organismal aging in a unified model.

## 2 Dietary restriction paradigms and nutrient-specific interventions

### 2.1 Caloric restriction

Caloric restriction (CR), typically defined as a 10%–40% reduction in total caloric intake without malnutrition, robustly extends lifespan and delays the onset of age-related functional decline in multiple species (Hofer et al., 2022). At the genetic level, CR reduces circulating glucose, insulin, and IGF-1, leading to attenuation of IIS and widespread transcriptional reprogramming favoring stress resistance and cellular maintenance.

### 2.2 Protein and amino acid restriction

Accumulating evidence suggests that dietary protein restriction (DPR) is a major determinant of DR-induced benefits. DPR lowers circulating IGF-1 levels, suppresses mTORC1 activity, and induces endocrine responses such as FGF21 secretion. Notably, restriction of individual amino acids can recapitulate many effects of global DR, highlighting the existence of amino acid–specific genetic sensing mechanisms (Kitada et al., 2019).

Key amino acids implicated in longevity regulation include branched-chain amino acids (BCAAs; leucine, isoleucine, and valine), methionine, threonine, serine, and arginine. BCAAs, particularly leucine, strongly activate mTORC1 via sensors such as Sestrin2. Methionine restriction alters one-carbon metabolism and epigenetic regulation, while serine and threonine availability influences nucleotide synthesis and redox balance. These findings underscore the importance of nutrient-specific genetic control in DR responses (Hofer et al., 2022; Kitada et al., 2019; Green et al., 2022; Ma et al., 2025; Upadhyayula et al., 2023; Green et al., 2023; Trautman et al., 2022; Hill et al., 2022; Lu et al., 2021; Solon-Biet et al., 2019; Hill et al., 2019; Longchamp et al., 2018; Cummings et al., 2018; Barcena et al., 2018).

### 2.3 Lipid restriction in dietary restriction

In addition to protein and amino acid availability, dietary lipid intake also induces metabolic adaptations. Lipid restriction improves insulin sensitivity and lowers circulating insulin levels, resulting in partial attenuation of insulin/IGF-1 signaling (IIS). At the cellular level, altered lipid availability influences mitochondrial substrate utilization and energy balance, promoting activation of AMP-activated protein kinase (AMPK) and suppression of mTORC1 activity (Lopez-Dominguez et al., 2015; Mut et al., 2021). Overall, lipid restriction modulates DR responses primarily by improving metabolic efficiency and reducing nutrient-induced stress, complementing the more direct lifespan-regulating effects of protein and amino acid restriction.

### 2.4 Temporal and modal dimensions of dietary restriction

**FIGURE 1:**
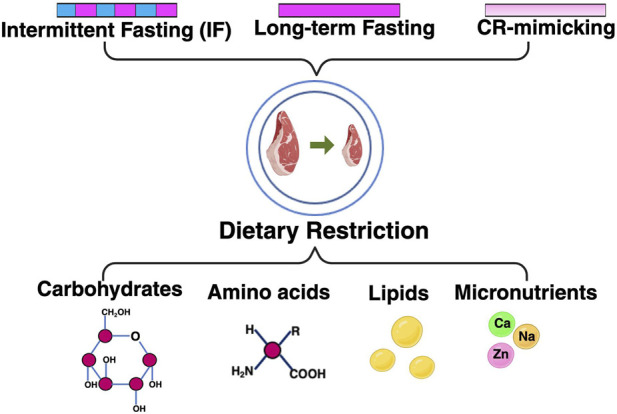
Overview of dietary restriction paradigms. Schematic overview of major dietary restriction (DR) paradigms classified by intervention timing and nutritional composition. Temporal strategies include intermittent fasting (IF), characterized by alternating feeding and fasting periods, and long-term fasting dietary restriction. Modal strategies include caloric restriction (CR)–mimicking approaches that reproduce key metabolic features of DR without nutrient deprivation. At the nutritional level, DR encompasses restriction of specific macronutrients, including carbohydrates, amino acids, and lipids, as well as selected micronutrients such as calcium (Ca), sodium (Na), and zinc (Zn). Despite differences in implementation, these DR paradigms converge on shared systemic metabolic adaptations, including reduced energy intake, altered hormonal signaling, and enhanced metabolic efficiency, collectively defining the dietary restriction state. Caveat: Note that while micronutrient restriction is included as a distinct dietary component, experimental evidence for its role in lifespan extension is currently less robust than that for macronutrient or caloric modulation, representing a burgeoning area for future investigation.

Beyond nutrient composition, dietary restriction (DR) can be classified based on the timing and mode of intervention (Figure 1).

Intermittent fasting (IF), including alternate-day fasting and time-restricted feeding, induces periodic suppression of insulin/IGF-1 and mTORC1 signaling, together with transient activation of AMPK (AMP-activated protein kinase) and autophagy. These cyclic metabolic shifts allow DR-like benefits without continuous nutrient limitation (Green et al., 2022; Duregon et al., 2021; Hwangbo et al., 2020).

In contrast, long-term DR establishes a chronic metabolic state characterized by persistently reduced anabolic signaling and stable transcriptional reprogramming, which is strongly influenced by age and physiological context (Waziry et al., 2023; Yang et al., 2016).

Beyond macronutrients, restriction of specific micronutrients, including Zinc, Calcium, and Sodium, can modulate metabolic homeostasis and stress responses (Sarmah et al., 2025; Chang et al., 2023; Liu et al., 2025; Shlisky et al., 2022; Song et al., 2009; Urban et al., 2024). Although micronutrient restriction alone rarely extends lifespan, it may influence DR-related pathways and modify organismal responses to dietary interventions.

Finally, dietary restriction mimetics, including rapamycin and metformin, activate key DR-associated pathways, such as mTOR and AMPK, without reducing food intake, offering complementary approaches for exploring DR mechanisms and improving healthspan (Green et al., 2022; Sharp and Strong, 2023; Hofer et al., 2021).

## 3 Core nutrient-sensing pathways and genetic regulators of dietary restriction

**FIGURE 2:**
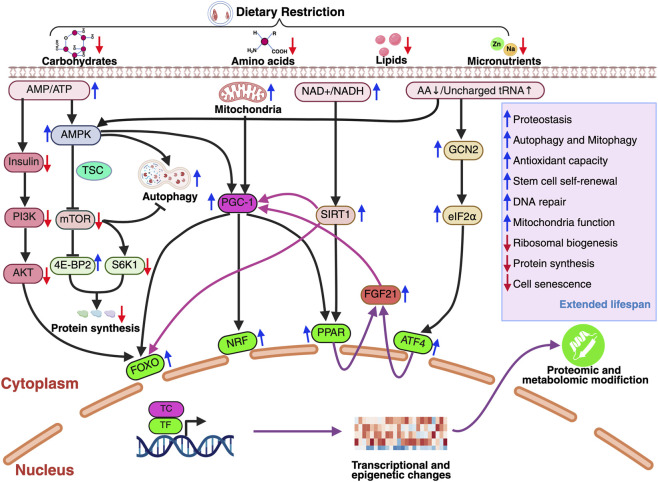
Core nutrient-sensing pathways engaged by dietary restriction. Dietary restriction (DR) alters nutrient and energy availability, including reduced glucose and amino acid supply, increased AMP/ATP ratios, and elevated NAD^+^/NADH balance. These primary metabolic cues activate conserved nutrient-sensing pathways. Reduced insulin and insulin-like growth factor 1 (IGF-1) signaling suppresses the phosphoinositide 3-kinase (PI3K)–protein kinase B (AKT) pathway, while energy stress activates AMP-activated protein kinase (AMPK). Together with amino acid limitation, these signals converge on the tuberous sclerosis complex (TSC) to inhibit mTORC. Amino acid deprivation also activates general control nonderepressible 2 (GCN2), leading to phosphorylation of eukaryotic initiation factor 2α (eIF2α) and induction of activating transcription factor 4 (ATF4). mTORC1 inhibition suppresses ribosomal protein S6 kinase 1 (S6K1) and eukaryotic translation initiation factor 4E-binding protein 2 (4E-BP2), reducing protein synthesis and ribosomal biogenesis while promoting autophagy. In parallel, increased NAD^+^ activates sirtuin 1 (SIRT1), which cooperates with AMPK to regulate Forkhead box O (FOXO), peroxisome proliferator-activated receptor gamma coactivator 1-alpha (PGC-1α), nuclear respiratory factors (NRFs), and peroxisome proliferator-activated receptors (PPARs). These integrated pathways enhance proteostasis, autophagy and mitophagy, mitochondrial function, and stress resistance, while limiting cellular senescence, ultimately contributing to extended healthspan and lifespan.

Dietary restriction (DR) initiates a hierarchical regulatory cascade that links changes in nutrient and energy availability to transcriptional reprogramming and organismal adaptation. At the first level, DR alters the abundance of primary metabolic inputs—including glucose, amino acids, lipids, and micronutrients—as well as intracellular energy and redox states, reflected by changes in AMP/ATP and NAD^+^/NADH (AMP: adenosine monophosphate, ATP: adenosine triphosphate, NAD^+^: nicotinamide adenine dinucleotide) ratios. These primary sensors activate conserved nutrient-sensing pathways and transcriptional regulators, which in turn drive coordinated downstream responses that promote cellular maintenance and longevity (Hofer et al., 2022; Green et al., 2022; Hwangbo et al., 2020; Hu et al., 2024; Fan et al., 2025; Fan et al., 2022; Zhao et al., 2024) (Figure 2).

### 3.1 Insulin/IGF-1 signaling as a systemic nutrient sensor

At the systemic level, reduced glucose and nutrient availability under DR lead to decreased insulin and insulin-like growth factor 1 (IGF-1) secretion. This reduction serves as a first-layer signal reflecting overall nutrient status. Attenuated insulin/IGF-1 signaling (IIS) suppresses PI3K–AKT activity, thereby activating FOXO transcription factors. Activated FOXO proteins translocate to the nucleus and induce gene expression programs involved in stress resistance, DNA repair, autophagy, antioxidant defense, and metabolic adaptation. Through this mechanism, changes in extracellular nutrient availability are translated into transcriptional responses that enhance cellular resilience. Genetic attenuation of IIS extends lifespan across multiple species, establishing IIS as a central conduit linking dietary inputs to longevity-associated gene regulation.

Genetic studies across model organisms reveal divergent roles of insulin/IGF-1 signaling in mediating dietary restriction (DR) effects on lifespan. In *C. elegans* and *Drosophila*, DR extends lifespan through mechanisms largely independent of the insulin/IGF-1 pathway; for instance, DR paradigms can further extend longevity in *daf-2* or *daf-16* mutants in worms, and in flies, lifespan extension by DR persists even in the absence of FOXO or without reductions in specific insulin-like peptides required for insulin signaling (Min et al., 2008; Houthoofd et al., 2003; Lakowski and Hekimi, 1998; Kenyon et al., 1993). In contrast, in mammalian models such as mice and rhesus monkeys, DR enhances insulin sensitivity, preventing insulin resistance and associated conditions like impaired glucose tolerance and type II diabetes, suggesting a more integrated role of reduced insulin/IGF-1 signaling in metabolic benefits (Stone et al., 2014; Bodkin et al., 1995; Kemnitz et al., 1994). These findings underscore the context-dependent and species-specific integration of insulin/IGF-1 signaling with nutrient availability and aging processes.

### 3.2 mTOR signaling as an integrative hub downstream of primary nutrient and energy sensors

The mechanistic target of rapamycin complex 1 (mTORC1) functions as a central second layer signaling hub that integrates inputs from multiple primary nutrient and energy sensors activated under dietary restriction (DR). The first sensing levels include reduced glucose availability, increased AMP/ATP ratios, diminished amino acid supply, and attenuated insulin signaling are transmitted to mTORC1 through distinct upstream pathways.

Reduced glucose availability and elevated AMP/ATP ratios activate AMP-activated protein kinase (AMPK), a key cellular energy sensor. Activated AMPK suppresses mTORC1 activity both directly and indirectly through phosphorylation and activation of the tuberous sclerosis complex (TSC), a critical negative regulator of mTORC1. Together, these glucose- and energy-dependent signals converge on mTORC1 to constrain anabolic growth under DR conditions.

Amino acid availability provides an additional primary sensing layer upstream of mTORC1. Reduced levels of growth-promoting amino acids are detected by amino acid–sensing mechanisms, leading to impaired mTORC1 recruitment and activation at the lysosomal surface. The combined effects of amino acid limitation, reduced insulin signaling, and energy stress result in robust suppression of mTORC1 activity during DR. Inhibited mTORC1 reduces ribosomal biogenesis and protein synthesis by its downstream effectors S6 kinase 1 (S6K1) and 4E-binding proteins (4E-BPs). Concurrently, inhibition of mTORC1 relieves repression of autophagy, promoting cellular recycling and quality control. mTORC1 serves as a key integrative node that translates metabolic inputs into coordinated transcriptional and metabolic outputs that support cellular maintenance and longevity.

The mTOR pathway represents one of the most conserved mediators of dietary restriction responses from yeast to mammals. Genetic or pharmacological inhibition of TOR signaling extends lifespan in yeast, worms, flies, and mice, and in multiple systems (Ferreira-Marques et al., 2021; Chen et al., 2019; Ma et al., 2015; McDaniel et al., 2011; Greer and Brunet, 2009; Hansen et al., 2008; Greer et al., 2007; Fan et al., 2026). These genetic epistasis experiments suggest that suppression of mTOR activity is a core mechanism through which DR promotes longevity. Importantly, mTOR integrates nutrient and growth signals to regulate protein synthesis, autophagy, and transcriptional programs, placing it at a critical junction between metabolic sensing and long-term cellular adaptation.

### 3.3 AMPK–sirtuin networks link energy and redox state to transcription

At the intracellular level, DR induces changes in cellular energy and redox balance, reflected by increased AMP/ATP ratios and elevated NAD^+^ availability. These parameters function as primary metabolic sensors that activate AMP-activated protein kinase (AMPK) and NAD^+^-dependent sirtuins, respectively.

AMPK activation signals energy stress and promotes catabolic processes, including autophagy and fatty acid oxidation, while directly inhibiting mTORC1. In parallel, increased NAD^+^ levels activate sirtuins such as SIRT1, which deacetylate transcription factors and transcriptional coactivators, including FOXO, PPAR and PGC-1α, thereby enhancing mitochondrial biogenesis and stress-responsive gene expression.

The regulatory role of these pathways is conserved across evolutionarily diverse taxa. In *C. elegans*, AMPK is indispensable for the lifespan-extending effects of DR (Greer et al., 2007). Similarly, in mammals, DR has been shown to elevate AMPK activity, which correlates with enhanced physiological performance, delayed functional decline, and extended survival (Ma et al., 2018; Ma et al., 2020).

Ultimately, this cross-species evidence reinforces the concept that DR-induced longevity is not merely a collection of isolated metabolic changes. Instead, it arises from a highly coordinated signaling hierarchy that converts transient nutritional cues into long-term transcriptional and epigenetic reprogramming, preserving proteostasis and mitochondrial integrity to support organismal resilience.

## 4 Transcriptional, epigenetic, and cellular effectors of dietary restriction

Dietary Restriction (DR) orchestrates a systemic longevity response that translates metabolic cues into a profound reprogramming of the epigenomic landscape (Hahn et al., 2017; Zhai et al., 2022). This transition is mediated by chromatin remodeling complexes that dynamically modulate DNA accessibility, alongside shifted enhancer and super-enhancer dynamics. Such regulatory hubs coordinate the expression of longevity-associated gene clusters, effectively establishing a transcriptional memory that supports long-term cellular resilience (Wakeling et al., 2009; Zhang W. et al., 2020). On a broader scale, DR maintains higher-order genome organization, preserving nuclear architecture and chromatin compartmentalization to prevent the global heterochromatin loss typically observed during aging. These structural safeguards are reflected in the slowing of epigenetic aging clocks, indicating that DR preserves a “younger” molecular state at the DNA level (Greer et al., 2025). While key transcription factors such as FOXO, ATF4, and PGC-1α serve as primary mediators (Longchamp et al., 2018; Greer et al., 2009; Kilberg et al., 2009; Kim et al., 2019; Palacios et al., 2009; Rodgers et al., 2005; Fan et al., 2019; Pan et al., 2017; Zhang S. et al., 2020), their functional impact is amplified through the reshaping of enhancer and super-enhancer dynamics. These regulatory hubs facilitate the coordinated expression of longevity-associated gene clusters, effectively establishing a transcriptional memory that supports long-term cellular resilience. This organism-wide coordination is further supported by endocrine factors such as FGF21 (fibroblast growth factor 21), particularly under protein and amino acid restriction (Hill et al., 2022; Hill et al., 2019). Emerging evidence also implicates the gut microbiome and nuclear pore-associated transcriptional regulation as critical mediators of these systemic DR effects (Zhou et al., 2025; Sbierski-Kind et al., 2022).

Ultimately, this multi-layered regulatory hierarchy drives key cellular effectors. Autophagy remains a central downstream process (Xia et al., 2024), facilitating the removal of damaged proteins and organelles to preserve proteostasis. Concurrently, DR induces mitochondrial remodeling through the coordinated regulation of biogenesis, dynamics, and mitophagy, which enhances metabolic flexibility and reduces oxidative stress (Savencu et al., 2021; Montefusco et al., 2021; Fontana et al., 2021). Finally, by attenuating proinflammatory signaling and reshaping immune cell composition, DR improves immune surveillance and sustains overall tissue homeostasis.

## 5 Dietary restriction confers tissue-specific and systemic benefits

**FIGURE 3:**
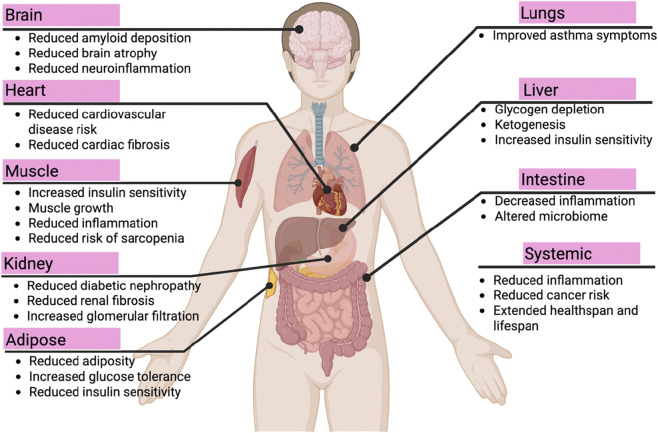
Downstream cellular effectors and systemic integration of dietary restriction responses. Dietary restriction (DR) promotes cellular maintenance through enhanced autophagy, proteostasis, mitochondrial function, and metabolic reprogramming. These cellular adaptations translate into tissue-specific benefits across multiple organs, including improved metabolic health, reduced inflammation and fibrosis, preserved organ function, and lowered disease risk. Collectively, these integrated responses contribute to extended healthspan and lifespan.

Dietary Restriction (DR) produces coordinated, tissue-specific benefits across multiple organ systems, reflecting the systemic integration of conserved nutrient-sensing pathways (Figure 3). However, the physiological response to DR is highly heterogeneous, characterized by tissue-specific transcriptional programs and varying degrees of engagement of the AMPK, mTOR, and sirtuin axes. For instance, while DR suppresses mTOR to promote autophagy globally, the metabolic priorities differ: the liver shifts toward gluconeogenesis and ketogenesis to maintain systemic energy, while skeletal muscle focuses on preserving mitochondrial flexibility and proteostasis (Suchacki et al., 2023).

Central to this systemic harmony is inter-organ communication mediated by a sophisticated network of endocrine mediators, neural signals, and hormonal inputs. A key player in this axis is FGF21 (fibroblast growth factor 21), which acts as a hepatokine that coordinates lipid metabolism in adipose tissue and modulates systemic insulin sensitivity. These endocrine cues, coupled with shifts in insulin and IGF-1 signaling, synchronize the metabolic rate and stress resistance across distant sites, including the brain and kidney.

In the brain and cardiovascular system, DR mitigates neuroinflammation and amyloid deposition while reducing fibrosis and cardiovascular risk through the local modulation of SIRT1-mediated stress responses (Fontana et al., 2021; Alkurd et al., 2024; Li et al., 2023; Lobo et al., 2022). Concurrently, metabolic tissues (liver, skeletal muscle, and adipose tissue) exhibit enhanced insulin sensitivity and reduced adiposity. In the kidney and intestine, DR-induced protective effects are further bolstered by modulations of the gut microbiota, which produce metabolites that reinforce intestinal barrier integrity and systemic anti-inflammatory states (Li et al., 2023; Jiang et al., 2023; Peng et al., 2024; Schmidt and Lorentz, 2021).

Notably, the efficacy of these systemic adaptations is subject to potential heterogeneity, influenced by age-, sex-, and context-dependent effects. Recent studies suggest that the onset of DR in early life versus middle age may engage different epigenetic memories, and sexual dimorphism in lipid metabolism can lead to divergent systemic outcomes between males and females (Harney et al., 2023). Collectively, these multi-organ adaptations, linked through a complex web of molecular and endocrine signaling, contribute to a reduction in systemic inflammation, lower disease risk, and the robust extension of healthspan and lifespan.

## 6 Limitations and context-dependence of dietary restriction

Despite its broadly beneficial effects, dietary restriction (DR) is not universally advantageous and exhibits strong context dependency. Excessive or prolonged DR can impair immune function, increase susceptibility to infection, and exacerbate frailty in aged populations (Fontana et al., 2021; Cunningham-Rundles et al., 2005; Cagigas et al., 2025). These detrimental outcomes often arise because DR acts as an intervention in which beneficial effects are confined to a specific physiological window, outside of this window, excessive restriction compromises immune competence, musculoskeletal integrity, and reproductive fitness.

Furthermore, DR can impair wound healing and tissue regeneration under chronic conditions, though transient or pre-injury restriction may improve survival following injury, highlighting a complex, non-linear relationship between nutrient availability and repair. Significant trade-offs also exist regarding reproductive function, where severe restriction suppresses fertility across species as an evolutionarily conserved mechanism to reallocate resources away from reproduction during nutrient scarcity. Finally, physiological responses vary significantly based on sex-specific factors—particularly in endocrine mediators like FGF21—and genetic background, necessitating individualized, context-aware approaches for the safe translation of DR into healthy aging strategies.

A consistent trend across comparative aging studies is that dietary restriction (DR) produces more robust and reproducible lifespan extension in short-lived organisms, such as yeast, *C*. *elegans*, and *Drosophila*, whereas in longer-lived species DR more reliably improves healthspan rather than maximal lifespan (Lakowski and Hekimi, 1998; Zhai et al., 2022; Di Francesco et al., 2024; Mattison et al., 2017; Mattison et al., 2012; Mair et al., 2003). In short-lived species, DR markedly delays mortality, likely reflecting strong suppression of growth-associated trade-offs and rapid engagement of stress-response and maintenance pathways. By contrast, long-lived organisms typically exhibit slower growth rates, lower baseline mortality, and extended developmental and reproductive phases, which may constrain the magnitude of additional lifespan extension achievable through further growth suppression. Long-term caloric restriction studies in non-human primates provide a critical bridge between short-lived model organisms and humans and illustrate this cross-species pattern. Two studies reported significant improvements in healthspan under caloric restriction, including enhanced metabolic health, improved cardiovascular function, and delayed onset of age-associated diseases, although only one study observed a significant effect on lifespan (Mattison et al., 2012; Colman et al., 2009). Importantly, differences between these outcomes have been attributed to variations in control diet composition, feeding regimens, genetic background, and husbandry conditions, underscoring the context dependence of DR effects even in closely related populations. Together, these primate studies reinforce the notion that while the core molecular mechanisms of DR are evolutionarily conserved, their phenotypic outcomes are shaped by life-history strategies and experimental context, highlighting healthspan extension as a particularly relevant and achievable endpoint for translational applications in humans.

## 7 Open questions and future directions in dietary restriction genetics

Despite substantial advances in understanding the genetic and molecular basis of dietary restriction (DR), important challenges remain. Key priorities include defining optimal timing, duration, and modality of DR interventions, clarifying genotype- and sex-specific responses, and establishing robust biomarkers to assess DR efficacy and safety. Dietary restriction mimetics targeting core nutrient-sensing pathways, such as mTOR, AMPK, NAD^+^ metabolism, and endocrine mediators including FGF21, hold translational promise but require careful evaluation of long-term effects. Moving forward, the integration of multi-omics profiling with genetic, physiological, and tissue-specific analyses will be important for translating DR mechanisms into precision nutrition and personalized health strategies aimed at improving healthspan and longevity.
